# Orientierungswerte in der isometrischen Kraftdiagnostik

**DOI:** 10.1007/s00132-024-04473-y

**Published:** 2024-02-20

**Authors:** Jan Schröder, Rüdiger Reer

**Affiliations:** Institut für Bewegungswissenschaft, Sport- und Bewegungsmedizin, Universität Hamburg, Turmweg 2, 20148 Hamburg, Deutschland

**Keywords:** Referenzwerte, Rumpfkraft, Kniestreckung, Kniebeugung, Funktionelle Quotienten, Reference values, Trunk strength, Knee extension, Knee flexion, Functional ratios

## Abstract

**Hintergrund:**

Isometrische Kraftdiagnostik sichert im Rahmen orthopädisch-physiotherapeutischer Interventionen die Qualität der Wirksamkeit von Therapie und Rehabilitation. Zur klinischen Einordnung bedarf es dafür Referenz- oder zumindest Orientierungswerte für jedes einzelne Kraftdiagnosesystem, da direkte Vergleiche problematisch sind. Ob das auch für funktionelle Quotienten gilt, ist bislang kaum untersucht. In dieser Arbeit werden für zwei Kraftdiagnosesysteme Orientierungswerte für die isometrische Maximalkraft der Rumpf- und Kniegelenkbeugung, sowie -streckung und die daraus errechneten funktionellen Quotienten vorgestellt und im Hinblick auf ihre Reproduzierbarkeit und Vergleichbarkeit geprüft.

**Methodik:**

In einer punktuellen Querschnittstudie wurden für 98 gesunde Erwachsene (47 Frauen, Alter 25,7±8,2 Jahre, BMI 23,3±2,6 kg/m^2^) Orientierungswerte (M, SD, Median, IQR, 5%- und 95%-Perzentile) für die isometrische Maximalkraft und funktionelle Quotienten der Rumpf- und Kniegelenkbeugung bzw. -streckung für zwei Diagnosesysteme (Myoline, Frei medical) ermittelt. Anhand von 20 Probanden (50 % Frauen) wurden gegenseitige Varianzaufklärung (r^2^) und Reproduzierbarkeit (ICC3.1, SEM, VK%) überprüft.

**Ergebnisse:**

Beide Messsysteme ermöglichen reproduzierbare Krafttests (ICC3.1 0,76–0,95), die Quotienten waren weniger reliabel (ICC3.1 0,62–0,92). Die gegenseitige Varianzaufklärung (r^2^) der Krafttests variierte stark zwischen 19 und 68 %; für die Quotienten zwischen 5 und 21 %.

**Diskussion:**

Die Testergebnisse der Krafttests von Myoline und Frei medical sind nicht miteinander vergleichbar, was insbesondere auch für die funktionellen Quotienten gilt. Jedes System für sich ist reliabel. Die gerätespezifischen auf das Körpergewicht relativierten Orientierungswerte sind für die Praxis nützlich.

**Zusatzmaterial online:**

Zusätzliche Informationen sind in der Online-Version dieses Artikels (10.1007/s00132-024-04473-y) enthalten.

## Abstract (en)

**Background:**

In the environment of orthopaedic rehabilitation, isometric strength testing is part of the monitoring in order to document the success of the therapy. For clinical applications, reference values, or at least orientation benchmarks, are needed for every single device, because of serious concerns for direct inter-device comparisons. According to functional ratios, there is only little literature covering comparability concerns. This study aimed to demonstrate reference values for two strength diagnosis systems for trunk and knee-joint flexion and extension as well as resulting functional ratios along with analyses of reproducibility and vice-versa comparisons.

**Materials and methods:**

In a cross-sectional design, reference values (M, SD, Median, IQR, 5 and 95% percentiles) of 98 healthy adults (47 females, age 25.7±8.2 years, BMI 23.3±2.6 kg/m^2^) were assessed for trunk and knee flexion and extension and the according functional ratios using either the Myoline or the Frei medical system. For a sub-sample of 20 persons (50% females), the mutual explained total variance (r^2^) and reliability (ICC3.1, SEM, VK%) were analyzed.

**Results:**

Both systems were shown to be reliable (ICC3.1 0.76–0.95), while functional ratios demonstrated a lower reliability (ICC3.1 0.62–0.92). For peak forces, the mutual total explained variance (r^2^) ranged between 19–68%, for functional ratios on an even lower level (5–21%).

**Conclusion:**

The resulting strength test values, and especially the related functional ratios, obtained with the two strength test devices are not comparable at all, but each device was shown to be a reliable tool. Distributions of body weight adjusted peak forces and functional ratios may serve as device specific benchmark values for strength testing in clinical environments.

Die Ausprägung der Muskelkraft und ihre Erfassung ist bedeutsam in der Diagnostik, aber vor allem im Monitoring von orthopädischen Rehabilitationsmaßnahmen. Im Gegensatz zur isokinetischen Kraftdiagnostik unterscheiden sich isometrische Kraftmesssysteme in ihren biomechanischen Anordnungen, sodass für jedes System spezifische Orientierungswerte notwendig sind, um Patientendaten einordnen zu können. Zu diesem Zweck werden in diesem Beitrag Orientierungswerte Gesunder für zwei unterschiedliche Messsysteme vorgestellt und die erforderlichen wissenschaftlichen Gütekriterien geprüft.

Seit Ende der 1950er-Jahre werden Zusammenhänge zwischen Rückenbeschwerden und Rumpfmuskelkraftdefiziten, aber auch Effekte von Rumpfkrafttraining auf Funktionsdefizite bei Rückenschmerzen untersucht [1–3]. Seit den 1980er-Jahren finden sich Arbeiten, die die Ausprägung der Rumpfmuskelkraft in allen räumlichen Dimensionen für Gesunde und Rückenschmerzpatienten erheben [4, 5]. Im deutschsprachigen Raum müssen die Grundlagenarbeiten von Achim Denner hervorgehoben werden, der nicht nur die wissenschaftlichen Gütekriterien geprüft, sondern auch umfangreiche Referenzwerte erarbeitet hat [6, 7]. Neben der isokinetischen Kraftdiagnostik spielt die isometrische Maximalkraftdiagnostik eine maßgebliche Rolle und ist fester Bestandteil der Qualitätssicherung in der Rehabilitation muskuloskelettaler Erkrankungen [8, 9]. Von daher muss sichergestellt sein, dass die erhobenen Kraftkennwerte nicht nur wissenschaftlichen Gütekriterien genügen, sondern auch vor dem Hintergrund von gerätespezifischen Referenzwerten interpretiert und eingeordnet werden können [10, 11]. Die Gerätespezifik muss auch dann beachtet werden, wenn oberflächlich betrachtet eine große Ähnlichkeit zwischen verschiedenen Kraftmesssystemen besteht, da die Anordnung von Standardisierungsmaßnahmen (Polsterungen, Haltegriffen, Hebellängen oder Winkelstellungen) erhebliche Auswirkungen auf die realisierbaren Maximalkraftkennwerte haben kann [10, 12].

Für das Universalkraftmesssystem Myoline (Diers, Schlangenbad, Deutschland) liegen Reliabilitätsanalysen und Orientierungswerte für die Rumpfmuskelfunktion – nicht aber für die Kniestreckung und -beugung – sowohl für die Allgemeinbevölkerung als auch für orthopädische Patienten vor [10, 11]. Für die Krafttrainings- und -diagnosegeräte der Firma Frei medical (Kirchzarten, Deutschland) gibt es hier noch Forschungsbedarf.

Ziel der vorliegenden Arbeit war es, nach Überprüfung der Vergleichbarkeit der beiden Krafttestsysteme und der Sicherung der jeweiligen Reproduzierbarkeit erste Orientierungswerte für die Frei-medical-Geräte zu entwickeln und für das Myoline-System um die Kniestreckung und -beugung zu erweitern, um sie der klinischen Nutzung zur Verfügung zu stellen.

## Methoden

### Design

Für die vorliegende Untersuchung wurde vorab eine Korrelationsstudie zur Bestimmung der Testgüte und Vergleichbarkeit durchgeführt, bei der am ersten Testtag in randomisierter Reihenfolge jeweils die erste Messreihe für die beiden zu vergleichenden Krafttestsysteme und am zweiten Testtag in der Folgewoche (gleicher Wochentag, annähernd gleiche Tageszeit) die zweite Messreihe durch immer dasselbe Untersuchungsteam ermittelt wurden. Für die Orientierungsdatenerhebung wurden weitere Probanden in gleichartig randomisierter Reihenfolge im Sinne einer punktuellen Querschnittstudie an nur einem Tag untersucht. Evidenzlevel 3.

### Stichprobe

Für die a priori Prüfung der Gütekriterien und gegenseitigen Varianzaufklärung wurden 20 gesunde Probanden (50 % Frauen) rekrutiert, die über die Studienziele und Untersuchungsmethoden informiert waren und ihre freiwillige Teilnahme attestierten. Für die Referenzwertdatenermittlung wurden weitere 80 gleichartige Probanden rekrutiert; bei einem Drop-out von zwei Personen in Teilen der Testungen konnten 98 Datensätze (47 Frauen, 51 Männer) der statistischen Analyse zugeführt werden (Tab. 1).

**Table 1:** 

		Alter (J)	KM (kg)	KH (m)	BMI (kg/m^2^)
Testgüteprüfung	Frauen (*n* = 10)	26,0	63,6	1,69	22,2
	SD	10,8	9,2	0,05	3,4
	Männer (*n* = 10)	29,8	79,4	1,80	24,4
	SD	10,7	11,7	0,03	2,8
	*p* (t-Test)	0,438	0,003	0,000	0,145
	Gesamt (*n* = 20)	27,9	71,5	1,75	23,3
	SD	10,6	13,1	0,07	3,2
Orientierungswertedatenbank	Frauen (*n* = 47)	25,6	63,4	1,69	22,1
	SD	8,7	7,7	0,05	2,3
	Männer (*n* = 51)	25,8	80,8	1,82	24,5
	SD	7,8	9,1	0,05	2,5
	*p* (t-Test)	0,930	< 0,001	< 0,001	< 0,001
	Gesamt (*n* = 98)	25,7	72,5	1,76	23,3
	SD	8,2	12,1	0,08	2,6

*BMI* Body-Mass-Index, *KM* Körpermasse, *KH* Körperhöhe

Bis auf den Ausschluss von akuten oder chronischen Beschwerden des Achsenskeletts und der Kniegelenke mussten bei dieser Klientel ohne Vorerkrankungen und Vorschädigungen keine weiteren Ein- oder Ausschlusskriterien formuliert werden.

### Testinstrumente

#### Myoline

**Figure 1:**
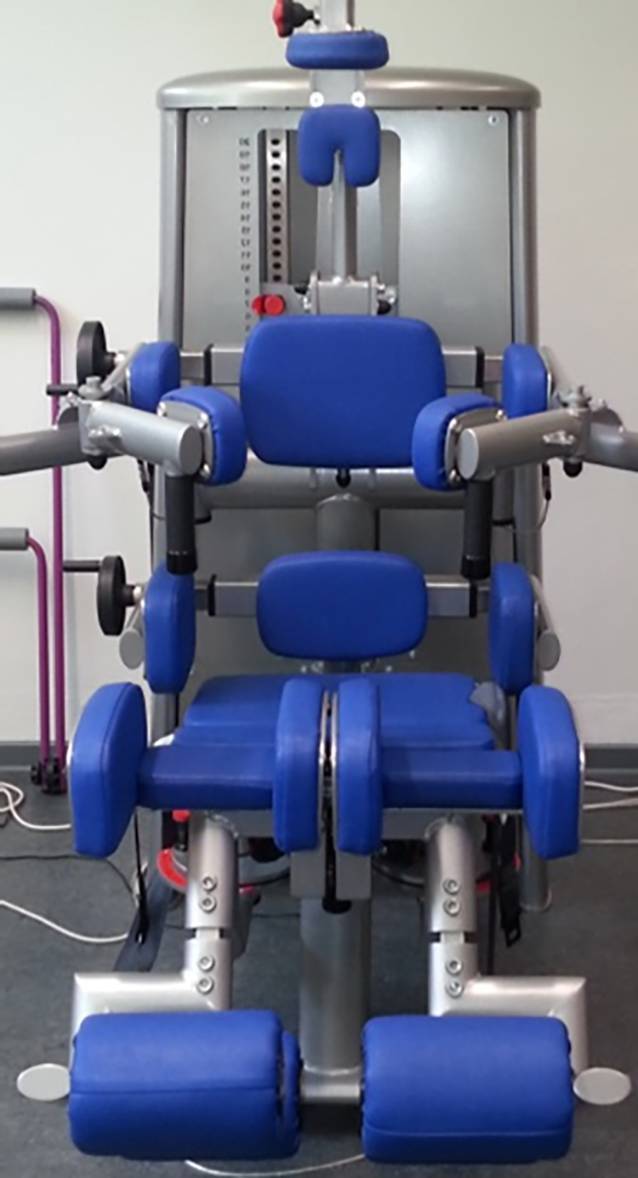

Die Rumpfmuskelkraft in der komplexen Rückenstreckung und Rumpfbeugung wurde im Sitzen bei etwa 90° Hüftwinkel unter standardisierten Bedingungen mithilfe des Messsystems Myoline (Diers, Schlangenbad, Deutschland) als isometrische Maximalkraft getestet (DMS-Kraftmessdose [Dehnungsmessstreifen]: 100 Hz, Filter: gleitendes Mittel über 0,3 s). Vor dem Hintergrund neuerer Standardisierungsüberlegungen wurde die Rumpfflexion und -extension ohne die Widerlageroptionen des Beinelements getestet, wie das für aktuelle Patientenreferenzwerte praktiziert wurde [10], wobei systematische Unterschiede zu früher publizierten Referenzwerten für Gesunde zu beachtet sind ([11]; Abb. 1).

Für die Kniestreckung und -beugung war der Ausgangskniegelenkswinkel jeweils 45°. Die interindividuell standardisierte Positionierung und Fixierung (Begrenzungsflächen, höhenverstellbares Schulterelement sowie Hüft- und Oberschenkelgurte) erlaubten eine zuverlässige Ermittlung der Kräfte in Extension und Flexion (Abb. 1**)**, zumal vor jeder Testung maximale Übungskontraktionen durchführt wurden, um die spezifischen Ansteuerungslernprozesse im Sinne der intra- und intermuskulären Koordination zu berücksichtigen [10, 11].

#### Frei medical

**Figure 2:**
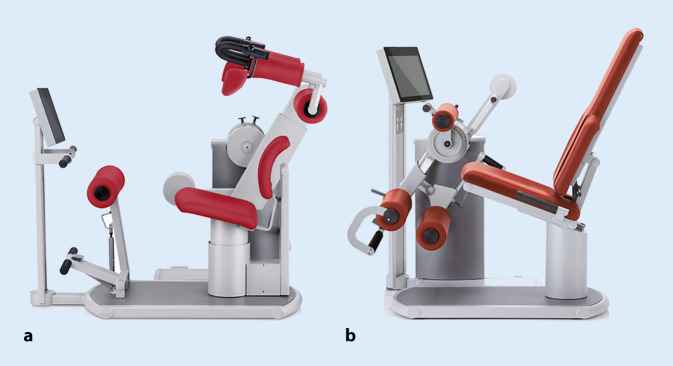

Die Krafttrainings- und -diagnosegeräte der Firma Frei medical (Kirchzarten, Deutschland) benötigen spezialisierte Geräte (Factum®), um die Rumpf und Knie umspannenden Muskelschlingen zu testen (Abb. 2). Die isometrische Muskelspannung wird mithilfe von firmenseitig kalibrierten Druckdosen und Scherkraftwegezellen auf Basis von DMS-Messstreifen (350 Ω) mit einer Abtastrate von 100 Hz erfasst (Fehlertoleranz ±5 % bei Null-offset vor jeder Messung). Die Testungen wurden einem Standardprotokoll folgend durchgeführt; Reliabilitätskoeffizienten wurden im Rahmen dieser Arbeit ermittelt (Tab. 2). Die Rumpfstreckung und Rumpfbeugung erfolgte nach Probekontraktion und mit progredienter Muskelanspannung aus einem Startwinkel von 10° Vorneigung. Ausgehend von einem rechten Winkel im Kniegelenk wurde für die einbeinige Kniestreckung ein Winkel von 80° und für die Kniebeugung von 50° gewählt.

**Table 2:** 

	Myoline Diers (N)		Frei medical (Nm)		Myoline × Frei medical		
	M	SD	M	SD	r	*p*_(r)_	r^2^
Rumpfextension	462,8	183,2	338,1	138,6	0,83	0,000	0,68
Rumpfflexion	161,6	73,5	187,9	57,8	0,70	0,001	0,50
Rumpf-Flex/Ex-Quotient (%)	37,3	17,6	58,3	14,5	0,39	0,091	0,15
Knie-Ext. (li.)	410,6	111,4	212,2	60,6	0,76	0,000	0,58
Knie-Ext. (re.)	388,3	104,2	211,6	56,1	0,67	0,001	0,45
Knie-Flex. (li.)	167,8	62,0	117,1	35,2	0,54	0,013	0,30
Knie-Flex. (re.)	174,6	69,4	117,0	35,0	0,43	0,056	0,19
Knie-Flex/Ex-Quotient (li.) (%)	40,8	9,4	56,0	14,9	0,22	0,342	0,05
Knie-Flex/Ex-Quotient (re.) (%)	45,4	13,3	55,4	8,3	−0,39	0,090	0,15
Knie-Ext.-Quotient li.-re. (%)	106,6	12,5	101,1	13,6	0,46	0,044	0,21
Knie-Flex.-Quotient li.-re. (%)	98,4	19,0	100,9	18,9	0,37	0,113	0,13

### Testprotokoll

Die Teilnehmenden wurden in der ersten Visite über das Forschungsvorhaben, die Ziele und Methoden aufgeklärt und gaben ihr schriftliches Einverständnis. Im Weiteren wurden die Probanden in zufälliger Reihenfolge in den Dimensionen Rumpfstreckung/-beugung und Kniestreckung/-beugung links und rechts in beiden Messsystemen getestet. Das isometrische Maximalkraftplateau sollte bei allen Testungen nach langsam progredientem Spannungsaufbau für 3–5 s gehalten werden.

Beim Myoline wurde das Multifunktionsgerät einmal für eine Person eingerichtet. Die Ermittlung der Rumpfstreckung/-beugung sowie der Kniestreckung/-beugung erfolgte nacheinander. Beim Myoline wurde die Muskelkraft des linken und rechten Beins in Streckung und Beugung jeweils simultan innerhalb einer Maximalkontraktion separat ermittelt. Die Myoline-Testungen wurden innerhalb von 10 min absolviert.

Beim Frei-medical-System gab es für die Rumpf- und Kniekrafttestungen jeweils spezialisierte Trainings- und Messgeräte, die dann auch jeweils für die Probanden eingerichtet werden mussten. Die Testungen der linken und rechten Kniestreck- und -beugekraft wurde separat durchgeführt, um für jede Extremität Einzelergebnisse zu erhalten, sodass z. B. die Symmetrie errechnet werden konnte. Die Testbatterie der Frei-medical-Geräte wurde innerhalb von 15 min absolviert, sodass die Probanden inkl. Rüstzeiten für insgesamt max. 30 min in Anspruch genommen wurden.

Die absoluten Kräfte (N) des Myoline-, bzw. Drehmomente (Nm) des Frei-medical-Systems und die auf das Körpergewicht relativierten Kräfte (N/kg) und Momente (Nm/kg) sowie die berechneten Quotienten (%) sowohl für die Rumpf- und Knietestungen (funktionelle Antagonisten: Flex/Ex) als auch für die Symmetrie (links und rechts) wurden der statistischen Analyse zugeführt.

## Statistische Methoden

Die Daten wurden parametrisch durch Mittelwert (*M*) und Standardabweichung (*SD*) beschrieben. Zur umfänglicheren Darstellung der Referenzwerte wurden zusätzlich auch die non-parametrischen Kennwerte Median und Inter-Quartilen-Range (IQR: Q25 %–Q75 %) und die 5 % und 95 % Randperzentile berechnet. Stichprobenunterschiede wurden nach Prüfung auf Normalverteilung (Shapiro-Wilk-Test) mithilfe des Student’s *t*-Test geprüft. Für die gegenseitige Varianzaufklärung wurde die einfache Pearson-Korrelation (*r*) mit Signifikanzlevel (*p*) und der Determinationskoeffizient (*r*^2^) berechnet. Bland-Altman-Analysen waren nicht indiziert. Zur Prüfung der „*relativen*“ Reliabilität wurde neben der Pearson-Korrelation und dem Determinationskoeffizienten auch der ICC_3.1_-Reliabilitätskoeffizient berechnet; als Maß für die „*absolute*“ Reliabilität wurde der Standardmessfehler (SEM = *SD* / √n) und der Variationskoeffizient (VK% = *SD* / *M* * 100) gemittelt für die Stichprobe bestimmt. Als Signifikanzniveau wurde *p* ≤ 0,05 festgelegt.

## Ergebnisse

Für Ergebnisvergleiche muss beachtet werden, dass sich die Maximalkraftkennwerte beider Systeme in ihrer Dimension unterscheiden; die Myoline-Krafttestungen werden in Newton und die Frei-Krafttestungen in Newtonmeter erhoben. Direkte Vergleiche der Absolutwerte waren somit nicht indiziert, Quotienten hingegen können gegenübergestellt werden.

Die Symmetriequotienten, die den intraindividuellen Vergleich der Kräfte der linken und rechten Kniestreckung und -beugung abbilden, ergaben für beide Testsysteme eine annähernde Symmetrie (98,4–106,6 %) der linken und rechten Körperhälfte, allerdings korrelierten die aus den Kraftwerten berechneten Symmetriekoeffizienten der beiden Systeme nur schwach, sodass sich eine gegenseitige Varianzaufklärung (*r*^2^) von lediglich 13–21 % ergab. Für die antagonistischen Funktionsquotienten (Flexion/Extension für Rumpf und Kniegelenk) wurde eine gegenseitige Varianzaufklärung (*r*^2^) von lediglich 5–15 % ermittelt – die mit den unterschiedlichen Messsystemen ermittelten Quotienten für die Rumpf- und Kniestreck- bzw. -beugekraft war somit weitgehend unabhängig voneinander (Tab. 2).

Die höchste gegenseitige Varianzaufklärung von 68 % wurde für die Erfassung der Rückenstreckkraft ermittelt. Die gegenseitige Varianzaufklärung beider Messsysteme für die Kniestreckung war größer (45–58 %) als für die Kniebeugung (19–30 %). Außer für die Rückenstreckung war die Fehlervarianz (unaufgeklärte Varianz) größer als die aufgeklärte Varianz, wenn Kraftkennwerte des einen Messsystems mithilfe des anderen Systems abgeschätzt werden sollten (Tab. 2).

Bei der Krafttestung mit dem Myoline-Testgerät lagen die Reliabilitätskoeffizienten der Rumpf- und Kniekraft zwischen ICC_3.1_ 0,76–0,93; die Reliabilitätskoeffizienten für die daraus berechneten Quotienten lagen darunter (ICC_3.1_ 0,62–0,76). Beim Myoline rangierte die absolute Reliabilität (SEM) für die Quotienten (%) zwischen 2,6 % und 4,8 %. Die Variationskoeffizienten variierten insgesamt zwischen 4,5 % und 12,1 % (Tab. 3). Bei der Krafttestung mit den Frei-System-Testgeräten lagen die Reliabilitätskoeffizienten der Rumpf- und Kniekraft zwischen ICC_3.1_ 0,88 und 0,95. Die Reliabilität für die berechneten Quotienten lag darunter (ICC_3.1_ 0,64–0,92). Für den Quotienten der funktionellen Antagonisten-Knietestung (Flexion/Extension rechts) wurde lediglich ein ICC_3.1_ = 0,38 ermittelt, was auf Ausreißerwerte innerhalb dieser Testungen hindeutet. Die absolute Reliabilität (SEM) für die Quotienten (%) rangierte zwischen 2,3 % und 4,0 %. Die Variationskoeffizienten variierten bei den Frei-System-Testungen insgesamt zwischen 4,1 % und 8,9 % (Tab. 3).

**Table 3:** 

		t1		t2		Test-Retest-Reliabilität					
		M	SD	M	SD	r tt	*p*_(r)_	r^2^	ICC_3.1_	SEM	VK%
Myoline Diers (N)	Rumpfextension	462,8	183,2	444,5	143,0	0,63	0,003	0,40	0,76	3,7	12,1
	Rumpfflexion	161,6	73,5	172,9	56,4	0,90	0,000	0,81	0,93	0,9	8,2
	Rumpf-Flex/Ex-Quotient (%)	37,3	17,6	40,4	12,2	0,66	0,002	0,44	0,76	3,5	12,2
	Knie-Ext. (li.)	410,6	111,4	398,9	107,7	0,80	0,000	0,64	0,89	1,7	6,3
	Knie-Ext. (re.)	388,3	104,2	370,5	105,8	0,73	0,000	0,54	0,85	2,0	8,0
	Knie-Flex. (li.)	167,8	62,0	161,5	52,0	0,68	0,001	0,46	0,80	1,1	9,1
	Knie-Flex. (re.)	174,6	69,4	167,0	61,9	0,83	0,000	0,69	0,91	1,0	9,4
	Knie-Flex/Ex-Quotient (li.) (%)	40,8	9,4	41,4	11,9	0,56	0,010	0,31	0,71	2,6	8,0
	Knie-Flex/Ex-Quotient (re.) (%)	45,4	13,3	45,9	14,1	0,54	0,014	0,29	0,70	3,2	10,0
	Knie-Ext.-Quotient li.-re. (%)	106,6	12,5	108,7	13,5	0,54	0,013	0,29	0,70	3,4	4,5
	Knie-Flex.-Quotient li.-re. (%)	98,4	19,0	99,5	18,5	0,45	0,044	0,21	0,62	4,8	6,5
Frei medical (Nm)	Rumpfextension	338,1	138,6	343,1	104,7	0,83	0,000	0,70	0,89	18,5	7,4
	Rumpfflexion	187,9	57,8	195,2	63,5	0,88	0,000	0,77	0,93	8,3	6,9
	Rumpf-Flex/Ex-Quotient (%)	58,3	14,5	58,3	15,0	0,77	0,000	0,59	0,87	2,6	7,1
	Knie-Ext. (li.)	212,2	60,6	213,6	52,3	0,92	0,000	0,85	0,95	6,4	4,1
	Knie-Ext. (re.)	211,6	56,1	213,5	56,9	0,86	0,000	0,75	0,93	7,9	5,3
	Knie-Flex. (li.)	117,1	35,2	124,0	34,3	0,96	0,000	0,92	0,98	3,4	4,6
	Knie-Flex. (re.)	117,0	35,0	121,7	30,8	0,79	0,000	0,62	0,88	6,3	8,1
	Knie-Flex/Ex-Quotient (li.) (%)	56,0	14,9	59,2	16,2	0,86	0,000	0,73	0,92	2,3	5,6
	Knie-Flex/Ex-Quotient (re.) (%)	55,4	8,3	58,9	14,2	0,27	0,245	0,07	0,38	3,8	8,9
	Knie-Ext.-Quotient li.-re. (%)	101,1	13,6	101,7	13,6	0,47	0,035	0,22	0,64	3,6	5,0
	Knie-Flex.-Quotient li.-re. (%)	100,9	18,9	103,6	26,5	0,83	0,000	0,69	0,88	4,0	5,5

*ICC*_*3.1*_ Intraklassen-Korrelationskoeffizient, *M* Mittelwert, *SD* Standardabweichung, *SEM* absolute Reliabilität, *VK* Variationskoeffizient

Die hier präsentierten Daten zum Systemvergleich (gegenseitige Varianzaufklärung) und zur Reproduzierbarkeit basieren auf den Absolutwerten. Die nachfolgend vorgestellten Orientierungswerte wurden vor dem Hintergrund des klinischen Anwendungskontexts auf die Körpermasse relativiert aufbereitet (Tab. 4). Für wissenschaftlich-akademische Nutzungen werden die Orientierungswerte auch unadjustiert in einem Supplement präsentiert (Tab. S1).

**Table 4:** 

			M	SD	5 %	Q25 %	Med	Q75 %	95 %
Myoline Diers (N/kg KG)	Frauen	Rumpfextension	0,6	0,2	0,3	0,5	0,6	0,7	0,8
		Rumpfflexion	0,2	0,1	0,1	0,2	0,2	0,2	0,6
		Rumpf-Flex/Ex-Quotient (%)	46	52	21	29	33	41	174
		Knie-Ext. (li.)	0,6	0,1	0,3	0,5	0,6	0,6	0,7
		Knie-Ext. (re.)	0,5	0,1	0,4	0,5	0,5	0,6	0,7
		Knie-Flex. (li.)	0,2	0,0	0,2	0,2	0,2	0,3	0,3
		Knie-Flex. (re.)	0,2	0,0	0,2	0,2	0,2	0,3	0,3
		Knie-Flex/Ex-Quotient (li.) (%)	45	16	26	35	41	49	83
		Knie-Flex/Ex-Quotient (re.) (%)	46	14	26	38	43	54	74
		Knie-Ext.-Quotient li.-re. (%)	102	12	82	96	100	109	123
		Knie-Flex.-Quotient li.-re. (%)	98	17	69	86	100	110	123
	Männer	Rumpfextension	0,7	0,2	0,3	0,5	0,7	0,8	1,0
		Rumpfflexion	0,3	0,1	0,2	0,2	0,3	0,3	0,4
		Rumpf-Flex/Ex-Quotient (%)	45	18	23	33	41	52	96
		Knie-Ext. (li.)	0,6	0,2	0,4	0,5	0,6	0,7	0,9
		Knie-Ext. (re.)	0,6	0,2	0,3	0,5	0,6	0,7	0,9
		Knie-Flex. (li.)	0,3	0,1	0,1	0,2	0,3	0,3	0,4
		Knie-Flex. (re.)	0,3	0,1	0,1	0,2	0,3	0,3	0,4
		Knie-Flex/Ex-Quotient (li.) (%)	46	14	23	35	47	54	72
		Knie-Flex/Ex-Quotient (re.) (%)	48	18	20	35	46	60	79
		Knie-Ext.-Quotient li.-re. (%)	104	16	78	94	103	114	128
		Knie-Flex.-Quotient li.-re. (%)	102	21	73	90	100	110	151
Frei medical (Nm/kg KG)	Frauen	Rumpfextension	4,0	0,8	2,6	3,5	4,0	4,7	5,3
		Rumpfflexion	2,2	0,5	1,3	1,8	2,1	2,6	3,1
		Rumpf-Flex/Ex-Quotient (%)	56	14	32	44	58	65	81
		Knie-Ext. (li.)	3,1	0,6	2,0	2,7	3,1	3,4	4,3
		Knie-Ext. (re.)	3,1	0,6	1,9	2,7	3,1	3,4	4,2
		Knie-Flex. (li.)	1,6	0,3	0,8	1,3	1,6	1,8	2,1
		Knie-Flex. (re.)	1,6	0,3	1,0	1,4	1,7	1,9	2,0
		Knie-Flex/Ex-Quotient (li.) (%)	51	11	32	44	51	57	71
		Knie-Flex/Ex-Quotient (re.) (%)	53	9	36	49	52	58	68
		Knie-Ext.-Quotient li.-re. (%)	101	14	81	91	100	108	132
		Knie-Flex.-Quotient li.-re. (%)	97	14	77	89	95	103	124
	Männer	Rumpfextension	5,5	1,1	3,7	4,8	5,5	6,2	7,5
		Rumpfflexion	3,0	0,5	2,1	2,6	3,0	3,3	3,9
		Rumpf-Flex/Ex-Quotient (%)	57	21	34	45	55	61	75
		Knie-Ext. (li.)	3,5	0,8	2,1	3,0	3,5	4,0	4,9
		Knie-Ext. (re.)	3,6	0,8	2,3	3,0	3,6	4,0	4,9
		Knie-Flex. (li.)	1,9	0,4	1,4	1,6	1,9	2,1	2,5
		Knie-Flex. (re.)	2,0	0,4	1,3	1,7	2,0	2,3	2,6
		Knie-Flex/Ex-Quotient (li.) (%)	57	15	42	48	53	62	104
		Knie-Flex/Ex-Quotient (re.) (%)	56	13	37	49	55	60	87
		Knie-Ext.-Quotient li.-re. (%)	97	13	71	89	96	107	119
		Knie-Flex.-Quotient li.-re. (%)	100	27	74	88	96	105	137

## Diskussion

Die Arbeit hatte die Zielsetzung, zwei Systeme zur Testung der Maximalkraft in der Rumpf- und Kniestreckung bzw. -beugung im Hinblick auf ihre Vergleichbarkeit (gegenseitige Varianzaufklärung) und vor dem Hintergrund ihrer Reproduzierbarkeit zu untersuchen, um im Weiteren Orientierungswerte für eine gesunde Vergleichspopulation zu präsentieren.

Als Hauptergebnisse müssen die niedrigen Korrelationen zwischen den Vergleichssystemen herausgestellt werden, die lediglich eine geringe Varianzaufklärung von 5 –21 % für die klinisch relevanten funktionellen Antagonisten- und Symmetriequotienten ergaben. Die mit den unterschiedlichen Messsystemen ermittelten Quotienten für die Rumpf- und Kniestreck- bzw. -beugekraft war somit weitgehend unabhängig voneinander; d. h. die ermittelten Quotienten dürfen im klinischen Alltag nicht als gleichwertig interpretiert werden – z. B. bei Patienten, die von einer zur anderen Einrichtung überwiesen und mit unterschiedlichem Equipment weiterbehandelt oder getestet werden (Tab. 2). Daraus darf nicht abgeleitet werden, dass es bei einem der Messsysteme an interner Validität mangelt. Aber die vorliegenden Daten unterstreichen, dass Messwerte und insbesondere daraus abgeleitete funktionelle Quotienten unterschiedlicher Messsysteme – vor dem Hintergrund geräte- und testungspositionsspezischer intra- und intermuskulärer Koordinationsmuster – nicht unkritisch als äquivalent oder gleichwertig betrachtet werden dürfen.

Im Hinblick auf die Reproduzierbarkeit konnten in dieser Untersuchung für die Frei-medical-Testsysteme (fast) durchgängig höhere Reliabilitätskoeffizienten ermittelt werden (Tab. 3). Die im Vergleich mit den absoluten Krafttestungen (Rumpfflexion, -extension, Knieflexion, -extension beidseits) niedrigeren Reliabilitätskoeffizienten für die funktionellen Antagonistenquotienten (Flexion/Extension) sowohl bei Myoline- als auch bei Frei medical erklären sich naturgemäß dadurch, dass schon geringfügige Abweichungen bei wiederholten Testungen der Flexion und Extension in der Weiterverrechnung zu Quotienten sofort zu deutlichen Unterschieden im Ranking innerhalb der jeweiligen Stichprobe führen, was sich dann in niedrigeren Reliabilitätskoeffizienten ausdrückt.

Zur Einordnung der vorliegenden Daten gibt es lediglich für das Myoline-System annähernd vergleichbare Untersuchungen aus der Vergangenheit und hier auch lediglich für die Rumpfkraft [10, 11, 13]. Für die Kniestreckung und -beugung fehlen sowohl für das Myoline-System als auch für die Frei-medical-Testsysteme vergleichbare Datenerhebungen, sodass mit der vorliegenden Arbeit eine Erkenntnislücke geschlossen wird.

Der antagonistische Funktionsquotient zwischen Rumpfbeugung und -streckung wurde in einer früheren Arbeit als 33,8 % beschrieben [11], was sich grob näherungsweise in etwa mit den aktuellen Daten der Gütekriterienvoruntersuchung deckt (37,3 %, *n* = 20) (Tab. 2). Allerdings weichen die Mittelwerte für Frauen (46 %, *n* = 47) und Männer (45 %, *n* = 51) in der größeren Stichprobe der Orientierungswerte (Tab. S1) deutlich von den vorliegenden Literaturdaten ab [11], was auf interindividuell stark variierende Rumpfkraftkompetenzen hindeutet, wie das für die daraus berechneten Quotienten schon früher für Patientendaten problematisiert wurde [10].

Im Vergleich zu den hier vorliegenden Absolutwerten für die nicht auf das Körpergewicht relativierte Maximalkraft (Tab. S1) wurden im Rahmen der vorliegenden Datenerhebung in der Rückenstreckung und in der Rumpfbeugung Orientierungswerte ermittelt, für die in früheren Publikationen (andere Baureihe Myoline, Nutzung des Beinelementwiderlagers bei Rumpfkrafttestungen) abweichende Werte angegeben wurden [10, 11]. Die Reliabilität der Rückenstreckkrafttestung in der vorliegenden Arbeit bleibt hinter den Koeffizienten früherer Untersuchungen zurück, wobei anzumerken bleibt, dass die Daten aus dem Jahr 2009 mit einer Vorläuferbaureihe des Myoline-Messsystems erhoben wurden [13]. Literaturvergleiche bleiben also für wissenschaftlich-akademische Vergleiche problematisch.

Für die ärztlich-physiotherapeutische Praxis können die hier vorliegenden Daten für eine klinische Einordnung genutzt werden, wenn das exakt gleiche Messsystem genutzt wird. Hierfür werden vor allem die non-parametrischen Kennwerte (Median, Quartile, 5 % und 95 % Perzentile) empfohlen. Interindividuelle konstitutionelle Unterschiede klinischer Patientengruppen sprechen für die Nutzung der auf das Körpergewicht relativierten Referenzwerte (Tab. 4).

## Ausblick

Orientierungsdatenbanken sollten für weitere Muskelgruppen erstellt werden, was in zukünftigen Studien zu bearbeiten wäre. Soweit es geräteherstellerseitig möglich ist, sollten dabei vergleichbare (Maximalkraft‑)Testausgangspositionen gewählt werden, um Einflüsse der intramuskulären Koordinationsmuster bei maximaler isometrischer Spannung zu berücksichtigen.

## Fazit für die Praxis

- Die Krafttestungen jedes Messsystems für sich betrachtet sind inhaltlich valide, aber die Ergebnisse sind nicht miteinander vergleichbar.
- Selbst die errechneten funktionellen (Antagonisten‑)Quotienten sind voneinander unabhängig und somit nicht vergleichbar.
- Vorteile Myoline: Zeitökonomie durch einmalige Patientenpositionierung für alle Tests. Simultane Ermittlung der Kniestreck- und -beugekraft separat für die linke und rechte Seite.
- Vorteile Frei medical: Die Daten werden physikalisch zutreffender als Drehmomente (Nm) unter Berücksichtigung der Hebelverhältnisse erfasst. Die biomechanische Anordnung der (Kraft‑)Testbewegung ist denen der möglichen Trainingsbewegung angenähert, sodass der abzubildende Trainingstransfer auf die Testung als hoch einzustufen ist.

### Supplementary Information





## Abkürzungsverzeichnis

BMI: Body-Mass-Index
DMS: Dehnungsmessstreifen
Ex: Extension
Flex: Flexion
Hz: Hertz
ICC: Intraklassen-Korrelationskoeffizient
IQR: Inter-Quartilen-Range
kg: Kilogramm
KG: Körpergewicht
M: Mittelwert
min: Minute
N: Newton
Nm: Newtonmeter
Ω: Ohm
*p*: Statistische Irrtumswahrscheinlichkeit
Q25: 25%-Quartil
Q75: 75%-Quartil
r: Korrelationskoeffizient nach Pearson
r^2^: Determinationskoeffizient (gegenseitige Varianzaufklärung)
SD: Standardabweichung
sec: Sekunde
SEM: Standardfehler des Mittels
VK%: Variationskoeffizient (%)

## References

[1] Morgan FP, King T (1957). Primary instability of lumbar vertebrae as a common cause of low back pain. J Bone Joint Surg Br.

[2] Flint MM (1958). Effect of increasing back and abdominal muscle strength on low back pain. Res Q.

[3] Cady LD, Bischoff DP, O’Connel ER (1979). Strength fitness and subsequent back injuries in fire fighters. J Occup Med.

[4] McNeill T, Warwick D, Andersson G (1980). Trunk strengths in attempted flexion, extension, and lateral bending in healthy subjects and patients with low-back disorders. Spine.

[5] Mayer TG, Smith SS, Keeley J (1985). Quantification of lumbar function. Part 2: Sagittal plain trunk strength in chronic low back pain patients. Spine.

[6] Denner A (1997). Muskuläre Profile der Wirbelsäule.

[7] Schifferdecker-Hoch F, Denner A (1999). Mobilitäts‑, Muskelkraft- und Muskel-leistungsfähigkeitsparameter der Wirbelsäule. Alters- und geschlechtsspezifische Referenzdaten. Man Med.

[8] Müller G (1999). Zur Evaluation von Funktionsstörungen an der Wirbelsäule. Strukturdiagnostik versus Funktionsdiagnostik. Man Med.

[9] Müller G, Hille E (1996). Muskuläre Dysbalancen im Rumpf – Möglichkeiten und Grenzen der klinischen und maschinellen Diagnostik in der Sportmedizin. Dtsch Z Sportmed.

[10] Pietsch A, Schröder J, Reer R (2021). Referenzwerte in der isometrischen Kraftdiagnostik. Orthopade.

[11] Schröder J, Braumann KM, Reer R (2014). Wirbelsäulenform- und Funktionsprofile. Referenzwerte für die klinische Nutzung bei Rückenschmerzsyndromen. Orthopade.

[12] Kulig K, Andrews JG, Hay JG (1984). Human strength curves. Exerc Sport Sci Rev.

[13] Schröder J, Reer R, Mattes K (2009). Biomechanische Diagnostik in der orthopädischen Praxis: Zur Zuverlässigkeit der Messung von Rumpfkraft und Haltung in der Behandlung von Rückenschmerzen. Orthop Prax.

