# Supplementary material for: Reference values for isometric strength tests
Source: Orthopadie (Heidelb). 2024 Feb 20;53(3):209–17. [Article in German] doi: 10.1007/s00132-024-04473-y (PMC10896767; doi:10.1007/s00132-024-04473-y)
Supplement: Supplementary file 1 [file 132_2024_4473_MOESM1_ESM.doc]

**Tab. S1 (Supplement)** – Orientierungswerte absolute Rumpf- / Kniekraft

|  |  |  | M | SD | 5% | Q25% | Med | Q75% | 95% |
| --- | --- | --- | --- | --- | --- | --- | --- | --- | --- |
| Myoline Diers (N) | Frauen | Rumpfextension | 376,8 | 104,0 | 168,0 | 317,6 | 375,3 | 444,1 | 544,9 |
|  |  | Rumpfflexion | 144,3 | 69,9 | 77,6 | 107,7 | 125,7 | 151,2 | 329,2 |
|  |  | Rumpf-Flex/Ex-Quotient (%) | 46 | 52 | 21 | 29 | 33 | 41 | 174 |
|  |  | Knie-Ext. (li.) | 349,3 | 80,9 | 195,8 | 296,1 | 360,4 | 400,5 | 475,7 |
|  |  | Knie-Ext. (re.) | 343,1 | 70,0 | 200,2 | 297,0 | 346,8 | 394,2 | 454,8 |
|  |  | Knie-Flex. (li.) | 148,9 | 40,5 | 85,4 | 123,9 | 144,9 | 162,4 | 243,5 |
|  |  | Knie-Flex. (re.) | 152,4 | 33,8 | 97,9 | 134,4 | 150,0 | 173,6 | 216,0 |
|  |  | Knie-Flex/Ex-Quotient (li.) (%) | 45 | 16 | 26 | 35 | 41 | 49 | 83 |
|  |  | Knie-Flex/Ex-Quotient (re.) (%) | 46 | 14 | 26 | 38 | 43 | 54 | 74 |
|  |  | Knie-Ext.-Quotient li.-re. (%) | 102 | 12 | 82 | 96 | 100 | 109 | 123 |
|  |  | Knie-Flex.-Quotient li.-re. (%) | 98 | 17 | 69 | 86 | 100 | 110 | 123 |
|  | Männer | Rumpfextension | 541,7 | 182,8 | 254,7 | 413,4 | 554,4 | 655,2 | 845,4 |
|  |  | Rumpfflexion | 222,0 | 69,2 | 116,3 | 176,0 | 208,0 | 259,2 | 356,8 |
|  |  | Rumpf-Flex/Ex-Quotient (%) | 45 | 18 | 23 | 33 | 41 | 52 | 96 |
|  |  | Knie-Ext. (li.) | 490,2 | 148,8 | 257,8 | 372,4 | 468,0 | 585,0 | 793,4 |
|  |  | Knie-Ext. (re.) | 479,7 | 149,0 | 259,0 | 390,1 | 484,5 | 545,1 | 786,2 |
|  |  | Knie-Flex. (li.) | 215,3 | 68,1 | 105,4 | 168,0 | 214,5 | 272,0 | 326,3 |
|  |  | Knie-Flex. (re.) | 218,9 | 76,3 | 91,5 | 158,0 | 216,0 | 288,0 | 348,6 |
|  |  | Knie-Flex/Ex-Quotient (li.) (%) | 46 | 14 | 23 | 35 | 47 | 54 | 72 |
|  |  | Knie-Flex/Ex-Quotient (re.) (%) | 48 | 18 | 20 | 35 | 46 | 60 | 79 |
|  |  | Knie-Ext.-Quotient li.-re. (%) | 104 | 16 | 78 | 94 | 103 | 114 | 128 |
|  |  | Knie-Flex.-Quotient li.-re. (%) | 102 | 21 | 73 | 90 | 100 | 110 | 151 |
| Frei medical (Nm) | Frauen | Rumpfextension | 256,1 | 61,0 | 151,6 | 217,0 | 242,0 | 287,0 | 369,4 |
|  |  | Rumpfflexion | 138,4 | 37,7 | 83,8 | 107,0 | 131,0 | 169,0 | 209,4 |
|  |  | Rumpf-Flex/Ex-Quotient (%) | 56 | 14 | 32 | 44 | 58 | 65 | 81 |
|  |  | Knie-Ext. (li.) | 194,4 | 36,1 | 127,4 | 171,0 | 195,0 | 211,0 | 266,8 |
|  |  | Knie-Ext. (re.) | 194,3 | 37,1 | 120,2 | 172,0 | 196,0 | 222,0 | 251,2 |
|  |  | Knie-Flex. (li.) | 98,5 | 22,9 | 52,0 | 83,0 | 100,0 | 110,0 | 145,8 |
|  |  | Knie-Flex. (re.) | 101,9 | 22,0 | 66,0 | 86,0 | 102,0 | 112,0 | 146,8 |
|  |  | Knie-Flex/Ex-Quotient (li.) (%) | 51 | 11 | 32 | 44 | 51 | 57 | 71 |
|  |  | Knie-Flex/Ex-Quotient (re.) (%) | 53 | 9 | 36 | 49 | 52 | 58 | 68 |
|  |  | Knie-Ext.-Quotient li.-re. (%) | 101 | 14 | 81 | 91 | 100 | 108 | 132 |
|  |  | Knie-Flex.-Quotient li.-re. (%) | 97 | 14 | 77 | 89 | 95 | 103 | 124 |
|  | Männer | Rumpfextension | 450,2 | 111,6 | 252,3 | 386,8 | 447,0 | 513,8 | 661,4 |
|  |  | Rumpfflexion | 240,4 | 46,8 | 165,4 | 210,0 | 244,0 | 270,8 | 318,8 |
|  |  | Rumpf-Flex/Ex-Quotient (%) | 57 | 21 | 34 | 45 | 55 | 61 | 75 |
|  |  | Knie-Ext. (li.) | 280,0 | 66,6 | 168,9 | 238,3 | 283,5 | 319,5 | 404,1 |
|  |  | Knie-Ext. (re.) | 288,9 | 62,5 | 184,6 | 248,8 | 293,5 | 330,0 | 405,1 |
|  |  | Knie-Flex. (li.) | 155,4 | 37,1 | 105,6 | 128,0 | 153,0 | 177,0 | 211,9 |
|  |  | Knie-Flex. (re.) | 158,6 | 33,8 | 105,2 | 129,5 | 157,5 | 183,0 | 215,3 |
|  |  | Knie-Flex/Ex-Quotient (li.) (%) | 57 | 15 | 42 | 48 | 53 | 62 | 104 |
|  |  | Knie-Flex/Ex-Quotient (re.) (%) | 56 | 13 | 37 | 49 | 55 | 60 | 87 |
|  |  | Knie-Ext.-Quotient li.-re. (%) | 97 | 13 | 71 | 89 | 96 | 107 | 119 |
|  |  | Knie-Flex.-Quotient li.-re. (%) | 100 | 27 | 74 | 88 | 96 | 105 | 137 |
